# A triple risk model for unexplained late stillbirth

**DOI:** 10.1186/1471-2393-14-142

**Published:** 2014-04-14

**Authors:** Jane Warland, Edwin A Mitchell

**Affiliations:** 1 Mothers, Babies and Families: Health Research Group, School of Nursing and Midwifery, University of South Australia, Adelaide, Australia; 2 Department of Paediatrics, University of Auckland, Private Bag 92019, Auckland 1142, New Zealand; 3 School of Nursing and Midwifery, Division of Health Sciences, University of South Australia, Adelaide 5001, Australia

**Keywords:** Stillbirth, Triple risk, Vulnerable fetus

## Abstract

**Background:**

The triple risk model for sudden infant death syndrome (SIDS) has been useful in understanding its pathogenesis. Risk factors for late stillbirth are well established, especially relating to maternal and fetal wellbeing.

**Discussion:**

We propose a similar triple risk model for unexplained late stillbirth. The model proposed by us results from the interplay of three groups of factors: (1) maternal factors (such as maternal age, obesity, smoking), (2) fetal and placental factors (such as intrauterine growth retardation, placental insufficiency), and (3) a stressor (such as venocaval compression from maternal supine sleep position, sleep disordered breathing). We argue that the risk factors within each group in themselves may be insufficient to cause the death, but when they interrelate may produce a lethal combination.

**Summary:**

Unexplained late stillbirth occurs when a fetus who is somehow vulnerable dies as a result of encountering a stressor and/or maternal condition in a combination which is lethal for them.

## Background

Unexplained late stillbirth – at or beyond 28 weeks gestation - is a devastating event. In high resource countries the prevalence rate for late stillbirth ranges between 2 and 5 per 1000 births and has decreased very little in recent years [1]. Furthermore, between one third and one half of all late term stillbirths are unexplained, that is a specific cause cannot be identified, even in high income countries where autopsy and/or placental pathological examinations are available the unexplained rate can still be around 15% [2].

Unexplained stillbirth is a difficult problem to study because of the paucity of clues. Furthermore it is probable that there is heterogeneity of many of the antecedent causes. However, if improvements in prediction and prevention of stillbirth are to be made, specific risk factors which are modifiable should be targeted. Clinical practice and observational studies primarily target maternal risk factors such as maternal smoking, obesity and medical conditions. Studies of stillbirth have also identified the importance of placental problems. Furthermore, IUGR is well recognised as a risk factor for stillbirth.

**Figure 1:**
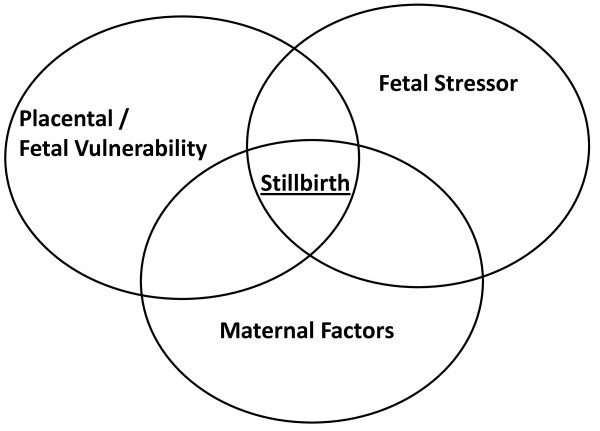
Triple risk model for unexplained late stillbirth.

However, few studies have focused on other factors that may impact on stillbirth risk such as fetal vulnerability and stressors which may heighten stillbirth risk. In this paper we propose a model which suggests that unexplained stillbirth occurs when three groups of factors (maternal, placental/fetal vulnerability and stressor) interrelate (see Figure 1).

The concept of a triple risk model is not new, indeed Sudden Infant Death Syndrome (SIDS) researchers proposed such a model to provide a framework for understanding SIDS risk. For example, Bergman [3] suggested that SIDS may not depend on any “single characteristic that ordains an infant for death” (p.210), but on an interaction of risk factors. Wedgwood [4] grouped risk factors into the first triple risk hypothesis consisting of general factors which could be responsible for raising the risk of death from any cause such as socio-economic factors, age-specific risks related to the victims developmental age, and precipitating factors including respiratory tract infection. Then Emery [5] raised the issue of susceptibility by hypothesizing that relatively minor conditions such as respiratory tract infection could, in susceptible babies, trigger a lethal cycle of events. In 1993 Rognum and Saugstad [6] developed a ‘fatal triangle,’ with groupings similar to those of Wedgwood; namely a vulnerable developmental stage, predisposing factors and a trigger event. Finally, Filiano and Kinney [7] presented arguably the best known triple risk model illustrating their hypothesis with a Venn diagram consisting of three concentric circles labeled vulnerable infant, critical developmental period and exogenous stressor/s with SIDS occurring at the intersection of all three circles. They proposed that but for underlying vulnerabilities the infant would not succumb to SIDS. The utility of this triple risk model is demonstrated by its ongoing use to illustrate SIDS research papers (e.g. [8]).

Here we suggest an unexplained late stillbirth triple risk model, illustrated in figure one, where such a stillbirth occurs as a result of an intersection of maternal factors and fetal vulnerability in the presence of a fetal stressor. Just as Filiano and Kinney did we also propose that but for underlying fetal vulnerability the fetus would not succumb to intrauterine fetal death. Here we discuss the specifics of our triple risk hypothesis.

## Discussion

### Maternal factors

There are many well-established epidemiologic maternal risk factors for stillbirth. Nulliparity, age, obesity, cigarette smoking, alcohol consumption have all been identified as potentially modifiable risk factors for stillbirth [2,9,10]. Common maternal disease such as gestational diabetes and hypertension as well as less common states such as antiphospholipid syndrome, lupus and heritable thrombophilias also have a well recognised association with stillbirth risk [11,12]. Additionally maternal infections both bacterial and viral can be catastrophic in terms of stillbirth risk especially in mid-gestation of the pregnancy [13]. Many women are overweight or obese at conception which predisposes them to increased risk of antepartum stillbirth [14]. Additionally excessive weight gain exposes the pregnancy to a range of poor outcomes [15] this occurs whether or not the woman started the pregnancy with a BMI in the normal range [16]. Whether the maternal factor is modifiable or not in our triple risk model these factors all sit within the ‘maternal’ factors circle.

### Fetal and placental factors

The abnormal fetus is known to be vulnerable to stillbirth especially mid-gestation (20–24 weeks) stillbirth [17]. It is also well recognized that twin pregnancy is at increased risk of stillbirth especially when complicated by twin to twin transfusion [18]. Other fetal factors associated with poor pregnancy outcome are sex of the fetus, via x-linked and other genetic factors [19]. Whilst it is well understood that the fetus with abnormal karyotype is vulnerable, as is the fetus with ‘intrinsic’ abnormalities [17]. There may also be as yet unexplored genetic or epigenetic factors responsible for fetal vulnerability for example when unexplained stillbirth reoccurs in families. Some unexplained stillborn and SIDS babies share common features found at autopsy especially abnormalities in the brain [20,21] suggesting that perhaps vulnerability to sudden unexplained death begins in utero.

Current research suggests that the fetus who is particularly vulnerable to late stillbirth is the fetus who fails to grow appropriately. Intrauterine growth restriction (IUGR) owing to placental insufficiency is identified in about 40–60% of stillbirths, also in otherwise unexplained stillbirths and highlights the probable role of placental pathology in stillbirth [10]. It is well known that the IUGR placenta is often abnormal in both structure and function e.g. [22,23]. Therefore, exploring underlying mechanisms for IUGR as well as early detection and effective management of fetuses who are at increased risk of developing IUGR, points the way for further research which could ultimately result in lowering stillbirth rates. Indeed a recent report [24] demonstrated a significant fall in stillbirth rates in areas of the UK which had adopted the use of customized growth charts to detect IUGR in pregnancy compared to those with low uptake, suggesting that intervention in this area is both possible and successful.

The fetus who slows or stops moving is also one who is vulnerable to stillbirth. Maternal perception of Decreased Fetal Movement (DFM) is reasonably common and can be benign, with 6–15% of women reporting at least one occasion of DFM to health professionals in the third trimester of pregnancy [25]. However, decreased fetal movement at or near the end of the pregnancy places the pregnancy at substantial increased risk of poor pregnancy outcome [25-27]. Distinguishing which fetus may be in trouble, from the fetus who is not, is therefore also an avenue for future research.

Placental dysfunction, and abnormalities also have a well-known association with poor pregnancy outcome [28-30]. It has also been suggested that the fetus at risk may stop movement to conserve energy in the presence of placental dysfunction [31]. In particular it has recently been reported that placentas from all pregnancies (irrespective of pregnancy outcome) with DFM had greater number of placental anomalies including areas of infarction, a higher density of syncytial knots as well as decreased villous vascularity and trophoblast area [32]. It is also understood that placental function diminishes as the pregnancy nears and goes over due [33]. Such research indicates the pivotal role that the placenta has in pregnancy outcome and may be an important factor impacting on fetal vulnerability in many stillbirths.

### Stressors

Some events are known to cause stillbirth such as cord prolapse, and ruptured vasa praevia [34]. Other events associated with increased risk of stillbirth include fetal–maternal haemorrhage and placental abruption. When these events occur they are associated with high rates of morbidity and mortality and provide a clear explanation for fetal death. Such events may be sufficient to cause death even in the presence of a healthy mother, placenta and fetus. Less severe events, such as nuchal cord, that would not cause death in the presence of a healthy mother, placenta and fetus may be sufficient to cause death in combination with a vulnerable fetus.

The kind of stressor we propose in our triple risk model may be more subtle than these dramatic events but in combination with the vulnerable fetus may result in death. There is emerging evidence which suggests that some stressors which may pose a threat to the fetus concern maternal sleep, in particular sleep position and duration [35,36], sleep state and architecture [37], sleep quality [38] and presence of sleep disordered breathing [39-42]. In and of themselves none of these can be lethal because sleep is an everyday occurrence in all pregnancies however, we propose that in the presence of fetal vulnerability events that occur during maternal sleep may be the tipping point for some fetuses.

One stressor for stillbirth may be reduced placental perfusion due to the mother lying supine whilst asleep. Indeed, it is biologically plausible that maternal left-sided sleep position may serve to protect the vulnerable fetus. Certainly it is well known that the left lateral position is the one that offers the best recovery position for the distressed fetus during labor [43] and it is also well known that the supine position in late pregnancy is associated with many hemodynamic changes caused by compression of the inferior vena cava and a resulting fall in cardiac output and placental supply [44,45].

It has been suggested that there are changes in sleep architecture during late pregnancy. For example REM (Rapid Eye Movement) sleep may be reduced with an increase in stage 1 (Non-REM) sleep and frequent awakenings from sleep [46]. The Apnea**-**Hypopnea Index (AHI) used to assess sleep apnea severity is more common during REM because muscles are more relaxed resulting in increased likelihood of airway obstruction. Therefore, it is possible that mothers who are deep sleepers may be more likely to have a higher AHI and thereby be at an increased risk of stillbirth. However, little is known as to whether or not these changes may be implicated in poor pregnancy outcome. Nevertheless, one study suggested that women who wake more than once overnight are at less risk of stillbirth than those who only wake once or not at all [35] perhaps suggests that the vulnerable fetus may not tolerate whatever is happening during a prolonged deep maternal sleep.

In addition to the already mentioned known impact of maternal obesity on poor pregnancy outcome overweight pregnant women are also at increased risk of developing sleep-disordered breathing [42]. The term sleep-disordered breathing (SDB) is used to describe a spectrum of abnormal breathing during sleep and is a rapidly emerging area of research when it occurs in pregnancy. Whether or not abnormal breathing during late pregnancy imposes the same risk in young otherwise healthy women as it does in overweight older population e.g. [47] is still largely unknown however, some recent studies have linked SDB with poor pregnancy outcome including hypertension and IUGR [16,42,48] Therefore exploring maternal sleep as a source of a range of stressors which may impact on the fetus and developing strategies to reduce this impact such as settling to sleep on the left and treating SDB in late pregnancy could be important steps in potentially protecting the vulnerable fetus from stillbirth.

Post –term pregnancy is known to be associated with stillbirth and therefore may also be a significant fetal stressor. As already mentioned this may be due to progressive uteroplacental insufficiency when the pregnancy progresses past term [33]. Animal and human studies have shown that various parameters of blood-gas and acid–base variables alter as pregnancy advances which may affect fetal growth and well-being [49-52]. Fetal lambs demonstrate reduced activity and increasing periods of quiescence as gestation approaches term [53] which may suggest that the post term fetus reduces movement to conserve energy in the presence of reduced utero-placental blood supply.

We also propose that there may also be as yet unknown, or less well known, factors at play in the ‘stressor’ circle where the causal pathway leading to fetal death is less obvious. Such stressors may not ordinarily be a problem for a well fetus but may become the tipping point for the vulnerable fetus. One such factor may be maternal hypotension. Whilst maternal hypotension is usually considered to be good in pregnancy there could be a link between hypotension and stillbirth [54-57]. It may be that this and other such stressors only become risk factors for stillbirth in the presence of a fetus who is vulnerable.

### The interplay between factors

Any of the slight reduction in stillbirth incidence in high incomes countries which has occurred in recent years has resulted from four distinct strategies [58]. These are effective management of risk factors such as alloimmunisation (Rh disease) and induction of labor for postdate pregnancies, effective management of maternal medical conditions such as hypertension and diabetes as well as increased intrapartum fetal monitoring and fetal surveillance and testing during pregnancy. However, so far proposed causal pathways leading to stillbirth do not enable potential victims to be identified, neither has an adequately discriminatory set of risk factors been devised.

It is likely that no single mechanism or causative pathway can explain stillbirth. Indeed most research has reported risk factors with odds ratios between 2 and 3 indicating that it is unlikely that any of these are the definitive cause of stillbirth, rather they might be additive or interact together resulting in a stillbirth particularly if the fetus is somehow vulnerable [59]. Further even in pregnancies exposed to many known risk factors most fetuses will not die, this also indicates that there are likely to be other factors at play in order for the fetus to succumb to stillbirth.

One of the roles of perinatal committees in high resource countries across the globe is to identify and classify stillbirth into an antecedent cause as well as collect and manage information about common clinical scenarios for future study and comparisons. The Wigglesworth [60], Whitfield [61] and PSANZ-PDC [62] systems are three of the more commonly used systems designed to facilitate this process. However, no classification system has been universally accepted. Furthermore the definition of stillbirth varies across countries, organisations and investigators, which make international comparisons of stillbirth rates difficult. This triple-risk model does not preclude the possibility that some stillbirths may be explained by a single antecedent cause with or without other contributory factors. However, this triple risk model is still useful especially in presenting what may have happened to parents, as we suggest that a fetus will die only if there is an interplay between risk factors and he or she is somehow vulnerable.

It should be noted that SIDS deaths did not decrease worldwide because a causative pathway for SIDS was discovered. Indeed it is still the case that a definitive causal pathway for SIDS is yet to be found, possibly because there are multiple causative pathways. SIDS deaths decreased worldwide because a means of simply and easily protecting vulnerable babies was discovered, namely settling all babies on their back to sleep [63,64]. Similarly if a means of protecting the vulnerable fetus is found then there may also be a resultant decrease in stillbirth prevalence. Also if stillbirth occurs because multiple variables are interacting, then preventive interventions to protect the vulnerable fetus will be effective in reducing stillbirth risk, irrespective of whether they relate to one cause or the other. We therefore suggest that research exploring ways of identifying and protecting the vulnerable fetus from stillbirth is key to reducing the unexplained late stillbirth rate.

## Summary

Our proposed triple-risk model can accommodate the complexity of a variety of intertwined factors that could work in concert to result in fetal death. Our model for late unexplained stillbirth is that it results from the intersection of: 1) maternal factors 2) fetal/placental factors, and 3) a fetal stressor. Death occurs only if all three factors intersect and only if the stressor and maternal factor match the specific vulnerability of the individual fetus. The latter explains why the same critical event and/or maternal factors are not always associated with stillbirth or even poor pregnancy outcome.

We suggest that unexplained late stillbirth occurs when a fetus who is somehow vulnerable dies as a result of encountering a stressor and/or maternal condition in a combination which is lethal for them. We propose that if a means of protecting the vulnerable fetus is found then this would essentially block the pathway to stillbirth in much the same way as laying all children supine protects the vulnerable infant from SIDS. The next advance in reduction of the rate of unexplained late stillbirth may require a more thorough understanding of the vulnerable fetus. Exploring factors which make the vulnerable fetus susceptible to stressors and specific maternal conditions and identifying a means to identify and protect the vulnerable fetus are therefore important areas for further research.

## Abbreviations

AHI: Apnea-hypopnea index; DFM: Decreased fetal movements; IUGR: Intrauterine growth restriction; PSANZ: Perinatal Society of Australia and New Zealand; REM: Rapid eye movement; SIDS: Sudden infant death syndrome.

## Competing interests

The authors declare that they have no competing interests.

## Authors’ contributions

JW drafted the manuscript. EM revised the manuscript critically for important intellectual content. Both authors devised the triple risk model. Both authors read and approved the final manuscript.

## Pre-publication history

The pre-publication history for this paper can be accessed here:

http://www.biomedcentral.com/1471-2393/14/142/prepub
